# Social ties in the Congo Basin: insights into tropical forest adaptation from BaYaka and their neighbours

**DOI:** 10.1098/rstb.2020.0490

**Published:** 2022-04-25

**Authors:** Adam H. Boyette, Sheina Lew-Levy, Haneul Jang, Vidrige Kandza

**Affiliations:** ^1^ Department of Human Behavior, Ecology and Culture, Max Planck Institute for Evolutionary Anthropology, Deutscher Platz 6, 04103 Leipzig, Germany; ^2^ Department of Comparative Cultural Psychology, Max Planck Institute for Evolutionary Anthropology, Deutscher Platz 6, 04103 Leipzig, Germany

**Keywords:** hunter–gatherers, mobility, inter-group relations, Congo Basin, cultural adaptation

## Abstract

Investigating past and present human adaptation to the Congo Basin tropical forest can shed light on how climate and ecosystem variability have shaped human evolution. Here, we first review and synthesize genetic, palaeoclimatological, linguistic and historical data on the peopling of the Congo Basin. While forest fragmentation led to the increased genetic and geographical divergence of forest foragers, these groups maintained long-distance connectivity. The eventual expansion of Bantu speakers into the Congo Basin provided new opportunities for forging inter-group links, as evidenced by linguistic shifts and historical accounts. Building from our ethnographic work in the northern Republic of the Congo, we show how these inter-group links between forest forager communities as well as trade relationships with neighbouring farmers facilitate adaptation to ecoregions through knowledge exchange. While researchers tend to emphasize forager–farmer interactions that began in the Iron Age, we argue that foragers' cultivation of relational wealth with groups across the region played a major role in the initial occupation of the Congo Basin and, consequently, in cultural evolution among the ancestors of contemporary peoples.

This article is part of the theme issue ‘Tropical forests in the deep human past’.

## 1. Introduction

Human evolution is classically portrayed as a story of adaptation to an emerging African savannah ecosystem. However, climatic and ecosystem variability is increasingly seen as the driving force in human evolution [1,2]. In this light, what adaptation to tropical forests can reveal about human evolution is relatively understudied. To address this gap, we first synthesize the archaeological, genetic and linguistic evidence for the expansion of humans into the Congo Basin and describe historical accounts of forager–farmer relations. Building from this evolutionary and historical perspective, we then describe the current social ties maintained by contemporary BaYaka foragers living along the Motaba river of the northern Republic of Congo. Throughout, we argue that the cultivation of relational wealth—that is, broadly, social ties that enable resource exchange and mutual assistance [3,4]—is central to forager adaptation. Particularly, links across forest forager communities in the Congo Basin, as well as trade relationships with neighbouring farmers and others (e.g. tradesmen, colonial administrators), support and have supported Congo Basin foragers’ dynamic subsistence practices over time in the face of shifting political, economic and ecological landscapes.

## 2. Becoming forest specialists: migration into the Congo Basin

Genetic work paired with palaeoclimatic reconstruction suggests that the last common ancestor of contemporary Congo Basin foragers^1^ and Bantu speakers is estimated to have lived approximately 70 000 ya in present-day Cameroon and Gabon [13–15]. Maternal gene flow dating to 40 000 years ago (ya) suggests these ancestral groups likely maintained ties as the foragers' ancestors migrated into the forest [14]. The earliest presence of modern *Homo sapiens* in the Congo Basin is estimated to be at least 40 000 ya [16,17]. Sites throughout the Congo Basin suggest continued occupation of the region since before the Last Glacial Maximum (LGM), around 20 000 ya [17,18]. The Lupemban stone tool industry is found in sites that, based on faunal remains, palynology and palaeoclimatological evidence, reflect humans living in a mosaic of tropical forests, woodlands and savannahs across the region [17,19,20]. The presence of the Lupemban industry in different ecologies suggests technological continuity from the savannahs into the tropical forests throughout the late Pleistocene into the early Holocene [20].

After moving east into what is today the interior of the tropical forest, the two major genetic branches of the contemporary Congo Basin forager population diverged around 20–30 000 ya, forming the Western (e.g. Aka, Mbendjele, Baka and Bakola) and Eastern (Mbuti, Efe and Twa) genetic sub-groups [14,15,21,22]. It is likely that these two populations diverged during relatively dry, cooler periods during which the forest was highly fragmented [23,24]. Despite growing geographical and genetic distance, connections between Eastern and Western groups likely persisted. For example, divergence times for Y-chromosome haplotypes between the Western and Eastern groups date to between 10 000 and 15 000 ya, long after the LGM (19–26 500 ya [25], cited in [13]). These findings suggest continued male-mediated gene flow, consistent with general patterns of greater male mating and exploration ranges among contemporary Congo Basin foragers [26,27]. The Western group later further divided around 3000 ya [15].

Congo Basin tropical rainforests likely posed unique ecological challenges to the ancestors of contemporary foragers [28–30]. The region is a complex mosaic of micro-ecosystems varying in soil composition and patterns of inundation, resulting in diverse and seasonal concentrations of edible plants and animals [31]. Forest foods can lack micro-nutrients essential to the human diet, such as iodine [32], and the forests are also host to many parasitic infectious diseases [33]. Evidence suggests there was positive selection on relevant biological functions and pathways among Congo Basin foragers that would have facilitated adaptation to these challenges, including those involved in immunology, thyroid hormone pathways (i.e. possibly in response to iodine deficiency [32,34]), thermoregulation, lipid metabolism and growth and development [35–39].

Bantu speakers likely migrated to the forest periphery from the savannahs of Northwestern Cameroon between 3500 and 5000 ya [40–42]. Historical linguistic analysis suggests that these migrants were savannah-dwelling foragers [43]. Bantu-speaking Iron Age farmers then replaced or displaced these Neolithic savannah dwellers some 2500 ya [40–42]. Linguistic analyses suggest that contact between Bantu speakers and Congo Basin foragers likely had a major impact on the cultures and economies of the latter groups. In particular, languages spoken by foragers prior to contact were mostly abandoned in favour of the Bantu languages spoken by these migrants, with only some ancestral forest-oriented vocabulary remaining today [44]. These cultural exchanges were dynamic, however, as indicated by the subsequent independent evolution of these languages within forager groups, and the fact that no foraging group today speaks the same language as the farming groups with whom they interact [12,45,46].

## 3. Maintaining inter-ethnic relationships: historical and ethnographic perspectives

Today, approximately 900 000 foragers live across nine countries in Central Africa [10], representing at least 15 ethnic groups [47,48] speaking 17 languages from six families [12,45]. Additionally, dozens of other ethnic groups, mostly Bantu-speaking farmers [49], subsist from shifting cultivation, fishing, commerce and a range of other practices [12,50,51].^2^ Throughout the Congo Basin, forager–farmer relations are multi-dimensional and highly variable [12]. While founded upon economic exchange, authors differently emphasize exploitation by farmers [58], forager–farmer solidarity in the face of outside forces [52,55,59] or forager agency as ‘hunters' of farmer resources [47,60]. These varying accounts nonetheless share the view that foragers maintain specialized and flexible subsistence knowledge and practices that have enabled both forager and farmer lifestyles throughout the region.

Pointing to the fact that sources of calories consumable by humans are scarce in rainforest settings, some have questioned whether foragers could subsist in the Congo Basin without access to cultigens from farmers [28–30,61]. Yet, contemporary foragers can and do live exclusively in the forest for extended periods of time [62–66]. Historical ecological data suggest that foragers themselves contribute to the propagation of wild yams [63,65]. Parts of *Dioscorea* tubers discarded during cooking lead to their dispersal, often in camps where light conditions from tree clearing favour growth [62]. Wild yams are also para-cultivated by reburying tuber parts with the intention of promoting their regeneration [65]. These practices highlight how forager activities and knowledge have likely shaped the availability and distribution of resources in the Congo Basin, which in turn can support forager communities [67].

Foragers are characterized by neighbouring farmers as the first inhabitants of the region [8,45–47,58]. Migrants, including Bantu speakers, historically depended on forager abilities and specialist knowledge to learn about their new forest environment [8,31,47,53]. Klieman [8] especially argues based on historical linguistic evidence that the foragers were essential to early farmer incursions into the forests, with different forager groups adopting roles as specialist procurers of forest products in exchange for iron, cultigens, and other material resources.

Farmer reliance on forager knowledge extended into the colonial period, when they served as default intermediaries between foragers and the states and corporations wishing to extract ivory, rubber, lumber and other commodities from the forest [9,46,53]. During this time, forager expertise and labour were critical to farmer political and economic survival, as the famers, who were sedentary and held land, were subject to taxation by colonial powers. In exchange, foragers gained economic resources, including not only cultigens but knowledge of their cultivation, and social connections to tradesmen and colonial administrators with whom they occasionally exchanged directly [9,44,51,68]. As such, foragers adapted their subsistence technologies to maximize their access to these resources. For instance, net hunting was adopted by foragers in the 1920s from Bantu-speaking farmers in response to colonial demands for duiker (*Cephalophus*) skins. The practice continued to be used opportunistically by some groups for decades, long after the market for pelts in France dried up [53,56,69,70].

## 4. Social links, diverse opportunities: a view from contemporary forest foragers

**Figure 1.**
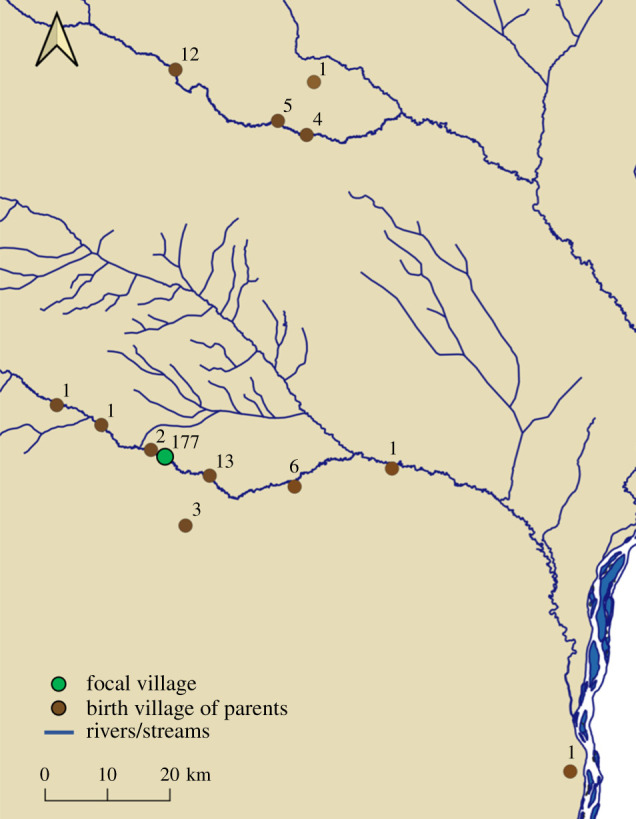
Regional connections to a Motaba village. In 2018, a sample of 124 individuals from the focal village along the Motaba (*indicated by a green dot*) reported the birth locations of their parents (*brown dots*). The numbers at each dot indicate the number of times the village was given as a birth location. This map shows inter-generational stability in the focal village but also reflects a snapshot of long-distance connectivity between communities in the region.

The nature of contemporary forager–farmer relations in the Congo Basin continues to be influenced by outside commercial, humanitarian and conservation interests in diverse ways [59,71–73]. Here, we describe how BaYaka foragers seek and integrate relational wealth into diverse livelihoods. We focus primarily on our work among several communities along the Motaba river in the Dongou District of the Likouala Department in the northern Republic of the Congo (figure 1). Along the Motaba, foragers identify as BaYaka/Aka^3^ [12,74–76]. From the upper Motaba downstream to the mouth of the river at Dongou, the largest farmer communities are Kaka, Bandongo and Bomitaba. Additionally, there are at least two villages inhabited by Ubangian speakers in the middle Motaba [12].

Along the Motaba, forager–farmer relationships are typically formalized within a fictive kinship framework. BaYaka are preferentially employed by farmer kin and perform their roles as family members during important events like births or deaths. Outside of the kinship framework, there are several BaYaka specialists hired by farmers to perform valued tasks. These include *nganga* traditional healers, who are sought for treatment or to identify the cause of an illness, and emcees who organize major gatherings such as funerals.

Via walking paths, the river and a growing system of roads, villages along the Motaba are linked socially and economically to each other, the rest of the region, the country and neighbouring parts of Central Africa. Below, we illustrate how mobility is woven into BaYaka social structure and culture, and how diverse, inter-group links contribute to their subsistence success.

### (a) Growing up mobile

Mobility is central to BaYaka lifestyles. Individual and family ranges depend on the availability of food resources and opportunities for social interaction. In turn, mobility structures how and from whom BaYaka learn as they grow.

As immediate-return mobile hunter–gatherers with limited food storage, BaYaka houses tend to be small and close together [77]. Most social and economic activities are performed outside. People tend to maintain close physical proximity with others throughout the day [77–79]. In this setting, young children are given ample opportunities to participate in economic activities and build social relationships beyond their own nuclear family. Infants are often turned outward by those holding them to direct the child's attention to the surrounding people and activities [80]. As children grow, adults may ask them to perform increasingly complex tasks (e.g. from fetching items across camp to carrying messages kilometres away) as a means of engaging them in daily routines ([81], also see [82]). A valued role for fathers in BaYaka culture is having children accompany them to the forest to learn during hunting and gathering trips [83,84]. With peers, children collaboratively learn subsistence knowledge and social norms essential to life in the forest and in society [85–88]. Most subsistence knowledge and skills are acquired before adolescence [89,90].

**Figure 2.**
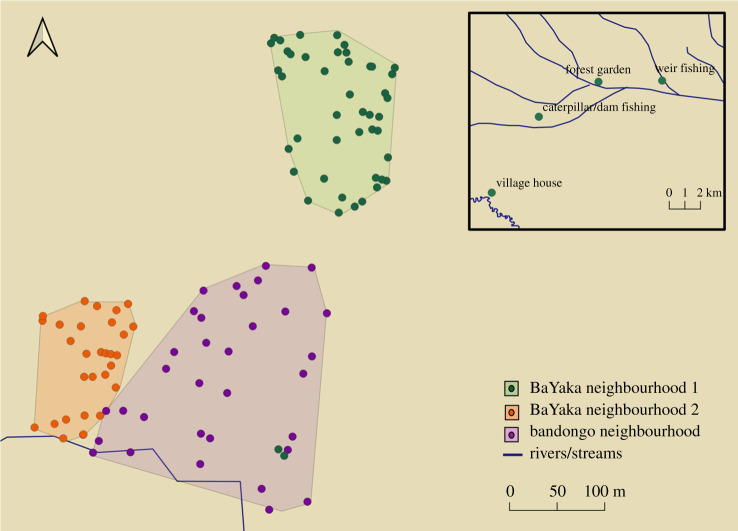
A multi-ethnic, spatially segregated village. This village has three neighbourhoods, two BaYaka and one Bandongo. Each point is a house marked from 2015 to 2018. Note that a BaYaka family from the northern community lived with a Bandongo family on their compound at the time for a period of labour exchange. *Inset*: One BaYaka household's seasonal residences and the location of their agricultural plot in the forest.

Residential groups are fluid, and the location of dwellings shifts in the context of seasonal mobility (figure 2). Many BaYaka along the Motaba spend approximately six months of the year in a multi-ethnic village setting. Villages are typically spatially segregated, with BaYaka and farmers residing in different neighbourhoods. BaYaka neighbourhoods can further be subdivided into hamlets that are loosely oriented around a core group of close kin (e.g. parents and their adult children, their affines and children). BaYaka regularly conduct day and overnight trips into the forest while settled in the village. In most villages, there are also two major periods of extended forest habitation: *kongo*, or caterpillar season from July to September, and *kombi*, or fish-weir season from October to December. In the forest, residential groupings are smaller and more dispersed with variable camp sizes. These camps are typically constituted by one or more families who also share a hamlet. The density of BaYaka houses seen in figure 2 reflects the social density of life in smaller forest camps.

The inset in figure 2 shows the geographical distribution of one family's seasonal camps. Of note is a forest garden 9.86 km from the village (in straight line distance). Most BaYaka have forest gardens, knowledge of which they say their ancestors acquired from farmers. Importantly, these gardens require little direct input. As gardens mature, they are more often used as ‘storehouses', with crops left to grow and reproduce on their own [91], a practice continuous with the process of encouraging wild *Dioscorea* yams to grow by clearing spaces in the forest [62,67]. The BaYaka family who planted the aforementioned forest garden did so with the specific intent of gaining independence from their farmer neighbours. Having this forest garden allows them to spend months away from the village while still maintaining access to cultigens.

Mobility is especially pronounced in adolescence and young adulthood, when finding a spouse from outside the local community is a priority [26]. The locations marked on figure 1 indicate the reported birth locations of the parents of 124 people living in one village (green dots) along the Motaba (data collected in 2018). The largest distance between where a parent was born and their adult child now lives is 82.4 km. As residence is multilocal [70,92], inter-community marriages give individuals and their families opportunities to build relational wealth. Indeed, traits that promote building the relational wealth of the community (e.g. sharing, welcoming) are seen as valued aspects of fatherhood among the BaYaka [83]. Mobility is central to the flow of knowledge throughout the Congo Basin, including of subsistence innovations [93–95], medicinal plants [88] and forest spirit dances [96].

Throughout history, Congo Basin foragers have used mobility as a strategy to avoid exploitative trade relationships, with individuals or groups moving deeper into the forest or searching for new farmer trade partners [46,97,98]. As the region has become more market-integrated, farmers continue to maintain trade relationships with the BaYaka, whose forest products are sold to urban centres in exchange for market goods or cash. The increasing pressure to school BaYaka children further impedes BaYaka mobility, with school calendars often conflicting with foraging activities [98]. Choosing to remain close to market towns has also been linked to decreases in traditional plant knowledge and preferences for traditional medicine [99], increases in wealth inequalities and a greater future-oriented time preference [100]. Still, while schooling may limit BaYaka mobility as it relates to forest activities, it may also place BaYaka in contact with new ideas and people [101]. Roads may increase BaYaka mobility, especially as it relates to labour opportunities. BaYaka in market towns continue to participate in the economy of forest spirits, with some reportedly investing up to four months' worth of wages into new dances [96]. These findings hint at the fact that while patterns may change, mobility will likely continue to be foundational to BaYaka identity and cultural practices.

### (b) Subsistence flexibility in a heterogenous ecosystem

There is considerable ecological variability along the Motaba river. Some villages are situated in Congolian Lowland Forests where streams do not typically go dry. Others are situated in a Western Congolian Swamp Forest ecoregion. These villages are located at higher elevation, have drier forest and are only seasonally inundated. Reflecting local subsistence adaptations to these ecoregions, villages along the Motaba practice differing fishing strategies [102].

Weir (*kombi*) and dam (*doka*) fishing are widely practiced fishing techniques along the Motaba. Unique to swamp forest ecoregions is *mosongo* fishing. *Mosongo* fishing uses permanent, human-dug fishponds and is practiced after *kombi* season has finished. Thus, for those with access rights to the ponds, *mosongo* can extend fishing seasons in the forest.

*Mosongo* has only recently been reported along the Motaba [102]. The geographically closest prior reports *of mosongo* are from several groups of Bantu C-20 language-speakers in the southern part of the ecoregion in the Cuvette some 300 km away [103]. There, inland fishing is a major economic activity, and pond fishing is widely practiced. It is possible that *mosongo* along the Motaba originated from traditional Bantu fishing in the southern floodplains and was spread to Likouala Bantu groups. This possibility is supported by the observation that Bantu-speaking people from Motaba villages, Impfondo, and Brazzaville travel to swamp forests to participate in pond fishing [102]. Typically, Bantu have access rights to multiple ponds inherited from their mothers or grandmothers.

While BaYaka labour at the Bantu ponds in exchange for a portion of fish, they also have their own ponds inherited through the maternal line [102]. These are typically located further into the forest. The fish collected for the visiting Bantu fisher–farmers are sold commercially, while in their own ponds, the BaYaka collect fish for their subsistence, or for sale at regional markets. Cash earned is primarily used to restock on flashlights, batteries and other commercial goods that they use throughout the year. *Mosongo* fishing demonstrates how inter-group relationships expose BaYaka to novel subsistence practices, and the ease with which BaYaka incorporate these into their cultural repertoire.

### (c) Hunting and global intersections

Consistent with historical accounts cited above, farmers along the Motaba claim their ancestors learned about the local ecology through the economic and social relations forged with BaYaka. BaYaka exchange products with farmers daily, including palm wine, *Irvingia excels* and *Treculia africana* nuts, and honey. They are also employed in making palm oil and corn liquor and as labour in gardens and for house construction. In exchange, farmers provide iron, salt, clothes and other agricultural and market products. Game meat is one of the most important forest products the BaYaka acquire for trade with their farmer neighbours [104].

As a result of taboos and the BaYaka's minimal access to cash, farmers are typically the exclusive owners of shotguns. As forest specialists, BaYaka are frequently tasked with hunting with these guns. Farmers will then sell bushmeat, earning a significant cash profit of 1000% or more per bullet. BaYaka hunters are typically paid with the ‘hunter's portion' of head, tail and entrails. BaYaka hunters make the best of this situation by keeping some animals while reporting to farmers that they ‘missed shots' [104]. Thus, BaYaka use their forest knowledge and skill as well as the Bantu dependence on these traits to their own advantage.

Due to conservation policies and logging, shotgun hunting may make BaYaka increasingly vulnerable [105]. Hunting endangered species is illegal, posing a major risk for hunters who can serve jail time for poaching. Road construction and the increased demand for bushmeat driven by logging are associated with increased sedentism and alcoholism among BaYaka hunters [53,71,106,107]. Because of logging activities, smaller and smaller sections of the forest are available for subsistence activities [108,109]. The likelihood of environmental degradation and game population decline has the potential to tip the balance from cooperation to exploitation of BaYaka shotgun hunters. At the same time, as game becomes increasingly rare and/or dispersed, shotguns may become one of the last viable technologies through which to successfully hunt [110]. In this context, the current rapid anthropogenic loss of the forest tests the strength of BaYaka mobility and relational wealth to buffer environmental changes.

## 5. Discussion

Biological adaptations to the pathogen-rich and relatively nutrient-poor ecology of the Congo Basin have undoubtedly been foundational to the continuous occupation of the region by forest foragers. In this paper, we have argued that alongside these biological adaptations, the cultivation of widespread inter-group relationships helped foragers develop subsistence practices adapted to the complex, closed canopy, humid tropical rainforest. We have further suggested that farmer expansion into the Congo Basin was dependent on forager knowledge of the forest. While we have focused here on the Congo Basin, social connections between distant and diverse groups are likely foundational to human adaptation more generally [4,111–116]. The movement of people, their genes, their things and their ideas across a wide expanse of Africa and beyond has shaped our species' evolutionary history [111,117,118].

Research has converged on a model of forager social structure in which groups have relatively low relatedness between their members as a result of the density of affinal kin [119,120]. Between groups, ties are maintained through marriage and close friendships [88,115]. These ties theoretically and empirically increase the efficiency of information flow [88,114,115]. The multiple, dynamic and large-scale linguistic shifts we have reviewed here further suggest that forest foragers have maintained a flexible and opportunistic strategy in relations with other groups, including with Bantu migrants, as part of a general strategy of building relational wealth and ensuring access to a broad range of resources and information across the challenging tropical forest landscape [8,46,53].

Despite extensive archaeological, historic and ethnographic evidence that diverse forager groups have formed a breadth of relationships with farmers, pastoralists and other non-foragers (e.g. for reviews: [121–123]), the adaptive role of these inter-group relationships has received relatively little attention [8,47]. Yet inter-group cooperation helps communities to withstand resource shortfalls and provides access to non-locally available resources [124–126]. Especially within biodiverse regions such as the Congo Basin tropical forest, niche specialization facilitates adaptation through trading informational (e.g. ecological knowledge, techniques), material (e.g. tools, forest foods, cultigens, imported market goods), cultural (e.g. meanings, beliefs, practices) and social (e.g. marriages, specialists, friendships) resources [127]. Such inter-group cooperation may help explain the observed linguistic diversity in highly biodiverse lower latitude ecologies in Africa and globally [128].

Foragers have always lived in shifting cultural and ecological landscapes. Whereas forest contraction and expansion drove forager mobility and inter-group exchange in the Congo Basin's past, the intertwined forces of market integration, schooling, logging, climate change and conservation efforts by outsiders are principal drivers today. Their effects on forager livelihoods vary considerably [73,106,129]. Continued research into Congo Basin forager patterns of mobility and interconnectedness in response to such forces has the potential to yield further insights into the micro-scale processes that led to human adaptation to tropical forests.

## Data Availability

The data used in this paper include GPS coordinates for locations of villages and people's houses to illustrate general spatial patterns. For reasons of privacy, we cannot make these data public. Requests for specific use of these data for research purposes can be made by contacting the corresponding author and will be judged on a case-by-case basis.

## Footnotes

1 Throughout, we choose not to use the terms ‘Pygmy’ or ‘Pygmies’ but instead use forager/foragers when writing generally, or the particular ethnonym of the group discussed as their name has been published (e.g., BaYaka, Baka). The term ‘Pygmy’ is in wide and continued use by academics across disciplines (e.g. [5–10]) as well as in the popular press. In the Congo Basin, the term is often used pejoratively by other ethnic groups to indicate ‘backwardness’ or being less than human, and can be used to admonish others for unacceptable behaviour *within* Congo Basin forager societies as well. However, it has also been reappropriated by many foragers for self-reference and connotes to them their unique identity vis-à-vis ‘non-Pygmies’ [11,12]. Our choice reflects how we would communicate about identity among our interlocutors in the Congo Basin.

2 We use the terms ‘foragers’ and ‘farmers’ or ‘fisher-farmers’ to contrast the two major groups found throughout the Congo Basin, each of which is constituted by peoples of multiple ethnicities. We recognize that these terms gloss over significant diversity in subsistence practices maintained by peoples in the region and that there is important overlap across the groups. Moreover, peoples' identities are shaped by more than how they acquire their food, such that these terms are not holistic labels [52]. Again, we use local ethnonyms whenever possible, but for purposes of generalization, we follow a tradition of work using these terms to compare these groups [7,47,53–57] and believe that at a minimum they connote the major ecological and economic relations of these societies that their core cultural features reflect and support.

3 These people have been referred to by both terms. We use ‘BaYaka’ after Lewis [11] as it is inclusive of the different forest forager groups differentiated by the foragers themselves, including Ngabo, Ngombe and Mbendjele.
